# Leveraging the process mining technique to optimize data preparation time in a database used as an automated data delivery center

**DOI:** 10.1016/j.mex.2025.103428

**Published:** 2025-06-12

**Authors:** Seyed Hossein Abrehdari

**Affiliations:** University of Tehran, Tehran, Iran; Institute of Geophysics and Engineering Seismology, National Academy of Sciences, Armenia; Amirkabir University of Technology (Tehran Polytechnic), Tehran, Iran; Institute of Seismology, University of Helsinki, Helsinki, Finland

**Keywords:** Big seismological database, Time optimization, Process mining, Unmanned tools, Big database, Scripts-layers

## Abstract

This study investigates the development and implementation of a seismic database utilizing process mining techniques. This data format is generated and stored in seismic centers, such as the U.S. Geological Survey (USGS). The study explored the various stages involved in the preparation, delivery, and processing of a database containing almost 900 earthquake waveform records (considered big data) by utilizing process mining techniques. The data were gathered from a region spanning 388,111.5 km², located between 44°–51°E and 38°–42.5°N, over the period from 1999 to 2018, and sourced from the USGS. The findings of this study indicate that the use of process mining methodologies decreases the time needed for database creation, including request, collection, preparation, and delivery, from 25 days with manual processing to approximately 8 days. In parallel, custom-built software scripts (computer codes) were deployed as unmanned tools to streamline the time-consuming phases of database creation. The idea presented in this study can help optimize the time for creating, storing, and delivering the database in seismological centers or other data centers, especially in an era where efficient management of large scientific datasets is increasingly vital. In total, process mining techniques were employed to analyze the workflow involved in creating a large database, including the steps of data request, preparation, and delivery.

## Review Highlights

• Technical diagrams (e.g., performance analysis) were depicted for process mining.
• An innovative approach was applied to optimize the time of process operations.
• A specialized map of the process mining was depicted using the particular event-logs.

## Graphical abstract

**Figure:**
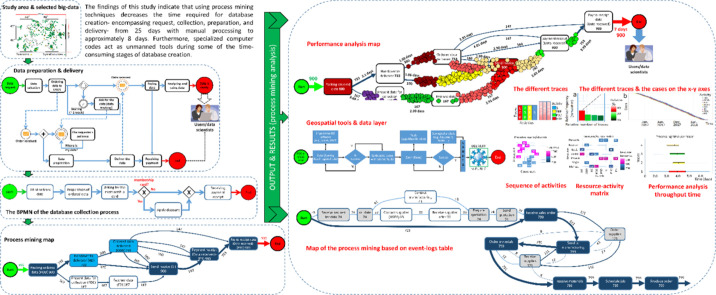

Specifications table

**Table:** 

**Subject area**	Computer Science
**More specific subject area**	Process mining and time optimization in big data processing management
**Name of the reviewed methodology**	Discovering the process mining diagrams
**Keywords**	Time optimization; Process mining; Unmanned tools; Big database
**Resource availability**	Data will be made available on request.
**Review question**	–

## Background

Many organizations can now gather extensive data to illuminate events and identify patterns that may lead to solutions. The management of time and the speed of data delivery and processing significantly impact the performance of scientists, PhD students, and specialists in this domain making it a crucial foundational aspect in the development of databases. This paper utilizes process mining techniques to analyse the execution history recorded in event-logs and describes description of the process from database creation through to delivery.

Despite these efforts to collect Big Data, event statistics in asset-intensive organizations continue to be alarmingly high, as the data often does not provide actionable insights. This paper develops a method to optimize time management in big data processing and applies big data science across various domains. It explores the potential of using process mining tools and techniques—a discipline dedicated to analysing process execution data—to generate new insights and improve the visualization of time optimization process data.

Through the generation of various process mining diagrams, this paper demonstrates how these tools can provide valuable and effective insights for time optimization in field of data processing by analysing process execution data from systems across knowledge-based, scientific, and non-scientific organizations. Specifically, the analysis presented here highlights the intrinsic complexity of database process mining, identifies performance issues, and uncovers related resources, thereby underscoring the necessity of simplifying the database delivery system. Inspired by the new perspectives and insights provided by process mining, it is anticipated that this approach will serve as a catalyst for accelerating data delivery, facilitating further research at the intersection of these disciplines.

## Method details

### Introduction

Process mining is an analytical technique that focuses on discovering, monitoring, and improving real business processes by extracting knowledge from event-logs generated by information systems. This method provides insights into how processes actually operate, rather than how they are perceived to function. By analyzing event data, organizations can visualize their workflows, identify inefficiencies, and enhance overall performance.

Process mining aims to discover, monitor and improve real processes by extracting knowledge from event-logs that are readily available in today’s information systems [1]. Over the last decade there has been a spectacular growth of event data and process mining techniques have matured significantly. As a result, management trends focused on process improvement and compliance can now leverage the benefits of process mining. The Key concepts of process mining encompass several important parameters related to real-world activities within organizations, categorized as follows:

Event-Logs: Every action within a business process leaves a digital trace in the form of event-logs. These logs contain timestamps and details about each activity, which are crucial for understanding the actual sequence of events. In essence, the starting point for process mining is an event-log. Each event in such log refers to an activity (i.e., a well-defined step within a process) and is associated with a particular case (i.e., an instance process). The events belonging to a case are ordered and can be seen as one “run” of the process. Event-logs may store additional information about events.

Process Discovery: This involves utilizing event-logs to automatically generate process models. By examining the data, organizations can reconstruct the actual flow of their processes, identifying any deviations from the intended workflow. A discovery technique takes an event-log and produces a model without using any a-priori information. For many organizations, it is surprising to see that existing techniques can indeed discover real processes based solely on example behaviors stored in event-logs.

Conformance Checking: This aspect evaluates whether the actual processes align with predefined models. It helps in identifying discrepancies and ensuring compliance with established standards. In this step, an existing process model is compared with an event-log of the same process. Conformance checking can be used to verify whether the reality captured in the log conforms with the model and vice versa.

Process Enhancement: The insights gained from process mining can lead to actionable recommendations for improving efficiency, eliminating bottlenecks, and optimizing resource allocation. Here, the idea is to extend or improve an existing process model thereby using information about the actual process recorded in some event-log. Whereas conformance checking measures the alignment between model and reality, this third type of process mining aims at changing or extending the a-priori model. For instance, by using timestamps in the event-log one can extend the model to show bottlenecks, service levels, and throughput times [[1], [2], [3]].

**Fig. 1:**
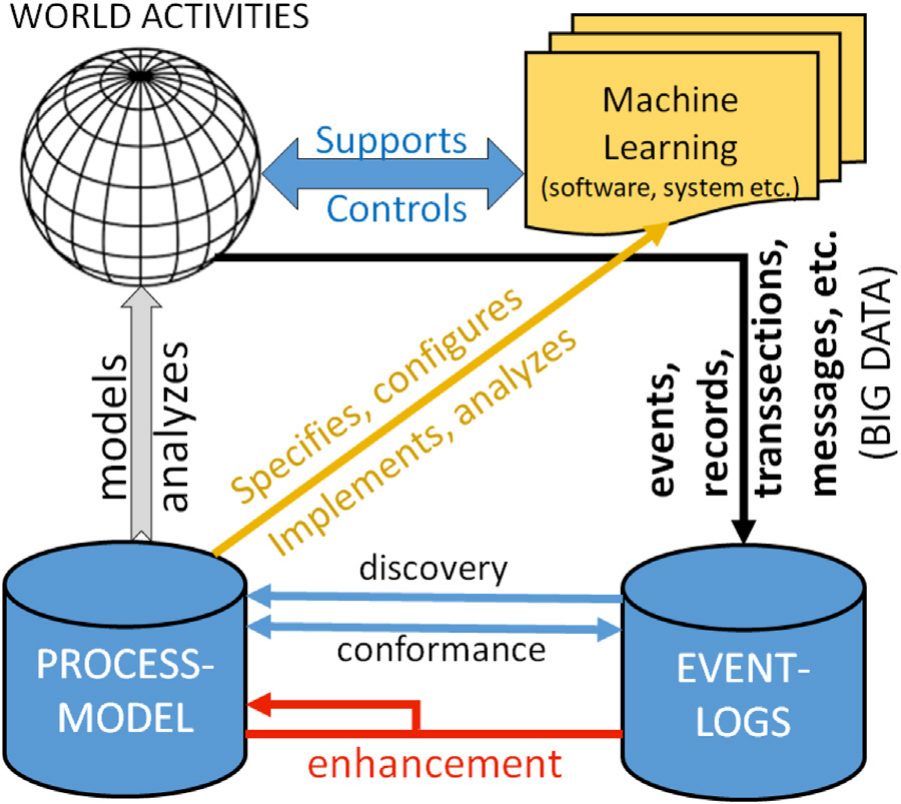
The three basic types of process mining are discovery, conformance, and enhancement. Figure retrieved from [4]. PowerPoint software was employed to enhance the clarity of the images.

Also, whenever possible, as shown in Table 1, process mining techniques use extra information such as the resource (i.e., person or device) executing or initiating the activity, the timestamp of the event, or data elements recorded with the event (e.g., the size of an order). Event-logs can be used to conduct three types of process mining as shown in Fig. 1 [4].

**Table 1:** A sample of a list of actions or event-logs related to Fig. 1, Fig. 2 in this study. The columns with red texts are not necessary for drawing the process mining map.

Commande_id	Activity_id	Resource_id	Timestamp	Depth	Magnitude
1	List of ordered data	Abry (PhdS)	2023–09–28 08:02:00	10	5.2
1	Preparation of ordered data	Abry (PhdS)	2023–09–28 08:25:10	14	2.3
1	Asking for the membership card	Abry (PhdS)	2023–09–28 08:36:39	8	2.1
1	Receiving payment receipt	Abry (PhdS)	2023–09–28 08:49:40	22	1.5
2	List of ordered data	Abry (PhdS)	2023–09–28 08:52:40	10	2.2
2	Preparation of ordered data	Abry (PhdS)	2023–09–28 09:12:29	14	3.3
2	Asking for the membership card	Abry (PhdS)	2023–09–28 09:23:54	8	3.1
2	Receiving payment receipt	Abry (PhdS)	2023–09–28 09:37:01	22	2.5
3	List of ordered data	Abry (PhdS)	2023–09–28 09:41:01	10	3.2
3	Preparation of ordered data	Abry (PhdS)	2023–09–28 09:50:28	14	2.3

According to research by [1,5], the advantages of process mining provide a strong foundation for organizational development. The *Transparency* benefit offers a clear understanding of how processes truly operate, facilitating more informed decision-making. The *Efficiency* benefit helps identify inefficiencies and areas for improvement, which can result in substantial cost savings and enhanced productivity.

Unlike traditional approaches, process mining utilizes data and *Real-Time Insights*, enabling organizations to swiftly address emerging challenges. The key strength of the *Broad Applicability* technique lies in its use across diverse industries-such as finance, healthcare, and telecommunications-making it a versatile tool for process optimization.

Studies by [6,7] highlight that, unlike Business Process Management (BPM), process mining is grounded in factual data recorded in event-logs combined with intelligent techniques for extracting knowledge. In essence, process mining is process-oriented rather than data-centric, truly intelligent as it learns from historical data, and firmly based on objective facts. Overall, process mining acts as a bridge between data science and operational management, providing organizations a powerful tool to enhance their processes -such as concurrency, selection, and iteration- and achieve improved outcomes (e.g., [1,8,6]).

This article not only introduces process mining in a simple way but also, by defining a set of guiding principles and listing important challenges, aims to serve as a guide for software developers, consultants, business managers, and end-users (scientists) to better understand the context of database preparation and delivery. It also proposes a seismic data analysis system developed by the IRSC that collects event-logs from a so-called database and analyzes the collected logs with a process mining. It should be highlighted that the process of storing and delivering the database is almost the same across seismological centers globally. In a short and concise sentence (nutshell), this study aims to describe and analyze: a) the data request from a data center, b) the steps involved in data preparation, and c) the delivery of a large database using process mining methods.

In fact, the primary objective (main goal) of this study is to reduce the time required for seismic data (databases) analysis by utilizing process mining techniques. Furthermore, process mining has been used in non-commercial contexts, with specialized computer codes acting as unmanned-tools during the time-consuming steps of database creation.

The remainder of this paper is organized as follows. Section 2 introduces an example of simple diagram of the process mining discovery, which serves as input for process mining to help readers better understand the concepts. It covers the necessity of studying the processes, event-logs of the processes, and process discovery. Section 3 describes the data. Section 4 presents the results and discussion of process mining and its related elements (e.g., scope, resource involvement, and performance) in this research. Finally, Section 5 concludes the paper.

## Methodology

### Discovering the process mining diagram in this study

A process is a sequence of activities performed to achieve a goal. Every business performs processes, whether formalized or not. Therefore, for complex business processes, it is essential to visualize how the organization or database functions, as this offers valuable insights into responsibilities, compliance analysis, performance forecasting, and more.

To achieve this, we begin by exploring the stages involved in requesting a large seismic database through process mining by presenting a straightforward problem, and then proceed to address the more complex aspects and fundamental layers of the database request process until it is delivered in the desired format.

### The main problem of the study

**Fig. 2:**
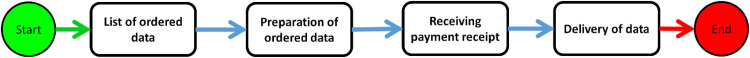
A sample of the process of database collection. PowerPoint software was employed to enhance the clarity of the image.

**Fig. 3:**
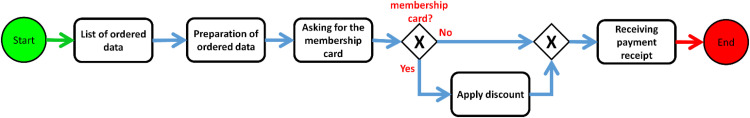
The process of database collection with discount (membership card). The tools available at bpmn.io, Disco, and bpmn-io on GitHub were used to draw the graph. PowerPoint software was employed to enhance the clarity of the images.

Abry is a PhD Student (PhdS) at the Institute of Geophysics, University of Tehran (IGUT), who plans to track and develop a database process as illustrated in Fig. 7. Initially, Abry (PhdS) attempt to address a simplified database process as illustrated in Fig. 2, which can be represented as follows:

To retain students and universities over the world (as customers for the purpose of article data delivery, if needed), Abry (PhD) has decided to implement a membership card that offers a discount. In this case, the process is as follows: 1- List of ordered data 2- Preparation of ordered data 3- Asking for the membership card 4- If provided (yes), applying the discount 5- Receiving payment receipt. This process is represented as follows:

In Fig. 3, there may be variations in the process, not all activities are executed each time the process is executed. As Fig. 2, Fig. 3 shows, the process discovery is the most prominent of process mining technique.

#### The necessity of studying the processes

Except some very simple processes (such as Fig. 2, Fig. 3), in the real world organizations and companies have much more complex processes (with hidden actions) that have to be done with many activities, actors, and also choices based on different criteria.

The execution of processes can be complicated by problems that require a different reaction that which is different from what is usually done (e.g., a wrong bank account number) or by actors who do not follow the process rules.

Organizations typically want to automate more of the tasks inherited from the past, thereby simplifying the process. However, humans prefer to automate processes that already work well to improve overall well-being. Meanwhile, our study aims to automate tasks to enhance speed and accuracy in the production, provisioning, and delivery of a seismic database. But how do you know if the automation process is effective and has a positive, constructive impact on customer service quality deviations, company costs, revenue, and other business metrics? Ultimately, the goal of skilled manpower behind these processes is to ensure that they evolve—whether through launching a new product or integrating new software.

#### Event-logs of the processes

Event-logging plays an important role as a starting point for process mining. Each activity (e.g., order number, item ordered, quantity) gives rise to the recording of information and the processes are born. These are the transactions stored in the databases and that humans are used to exploiting.

To create and study an event-log, the majority of companies and organizations work with a systematic information system (such as software for order taking, production initiation, and invoicing). By collecting all the information on each transaction corresponded to the event (such as creating and modifying an order), which contains information such as the date and time of the event, the person who performed it, they generate this event-log.

Manyika et al. [1] state in their study that digital event data is everywhere- in every sector, economy, organization, and home-and will continue to grow exponentially. The omnipresence of such data allows new forms new approaches to process analysis-based on observed facts rather than hand-made models-with event-logs serving as the foundational starting point for process mining.

Let’s go back to the case of the IGUT data processing section. Abry (PhdS) tracks all actions performed in the processing section and produces an event log as presented in Table 1. Each row of the table represents an event, i.e., an action performed related to an activity. We distinguish an event from an activity because an event is unique, but an activity can be repeated (e.g., repeating membership card or recording an event).

**Fig. 4:**
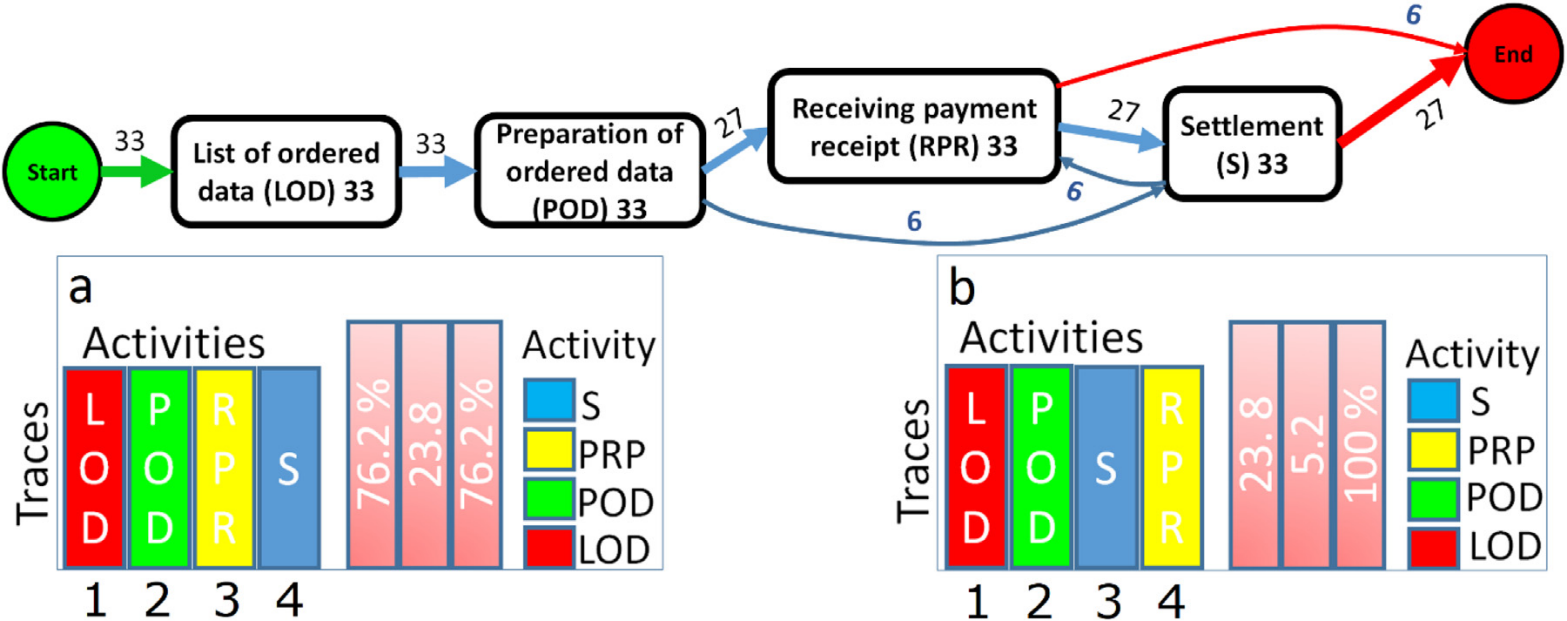
Map of the process (above). The tools available at bpmn.io, Disco, and bpmn-io on GitHub were used to draw the graph. PowerPoint software was employed to enhance the clarity of the images; a-b. The different traces left by the process, i.e. all the different sequences of activity that the process has taken, as well as their relative and cumulative frequencies.

From Table 1, it can be seen that each line is referenced by the command_id number, which serves as the identifier of the process instance. Thanks to this unique identifier, we can link the sequence of activities and reconstruct the execution of the process for this order. Event-logs make it possible to study a process from a huge volume of events, which is the domain of process mining. Using these events, it is possible to recreate the process as it actually happened, taking all events into account. By visualizing all process activities and different paths based on real data, the process can then be mapped (see Fig. 4).

Process of Fig. 4 (a-b) shows that the reality is a little more complex. In 23.8 % of cases, Abry (PhdS) does not follow the theoretical process. The figure mentioned shows the different traces left by the process, i.e. all the different sequences of activities that the process has followed, along with their relative and cumulative frequencies.

Also, it is possible to control what happens after Abry (PhdS) sets up the real program. In this case, starting from the event-log for this period, can display the map of the actual process (e.g., deviations) corresponds to the theoretical process. This will be omitted here to avoid lengthening the study and generating an excessive number of output images.

#### Process discovery

One of the functions of process mining is to discover the theoretical process from the event-log. This subsection runs parallel to 2.2.2, "Event-logs of the processes," with both sections complementing and expanding upon each other. Usually, the modeling of an existing process is manual and involves interviewing with various stakeholders which models a process based on what actors have explained and often results in an idealized theoretical process that does not reflect reality.

In the process mining literature, two common methods of Petri nets or Business Process Model and Markup (BPMN) are used to draw process models [9]. The process discovered by the algorithm from the event-log corresponds well to what we expected to find (equivalent to the process in Fig. 3).

Thus, by replaying the event-logs (Table 1) with the Petri nets, it is possible to automatically detect missing or remaining problematic tokens. This allows to focus directly on the instances that are not compliant (i. e., something that could not be done manually, on a process that has thousands or hundreds of thousands of instances as is the case with most companies).

## Data

**Fig. 5:**
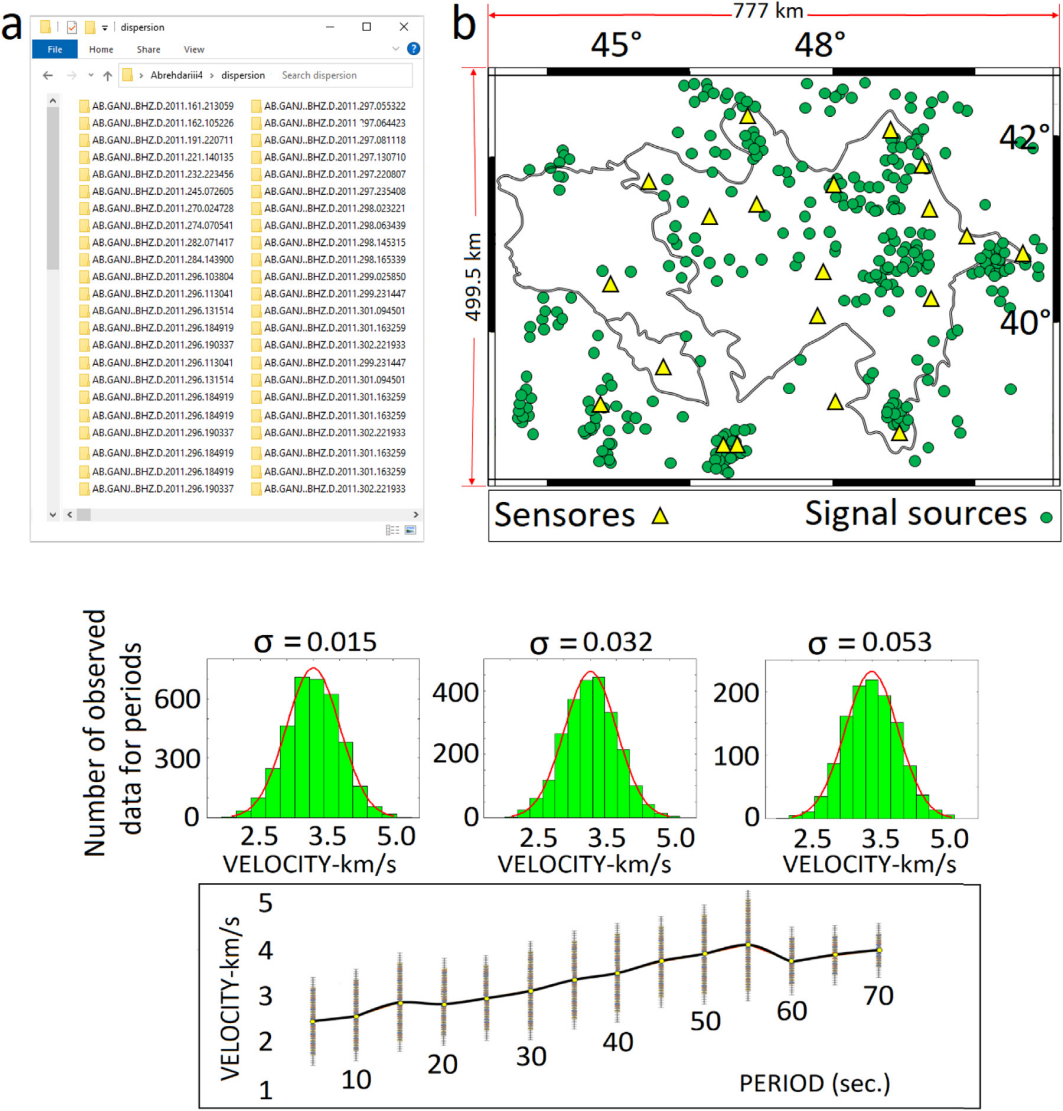
The study area; a. An example of a data format; b. The seismic signals are spread across an area of 388,111.5 km² (green circles). The map was generated using the Generic Mapping Tools (GMT) software. Fig. 5 histograms illustrate the distribution and the normal fit of the probability density function of the observed data across different periods (as shown above). The distribution of the input data is represented by crosses, while the bold black line indicates the average. The figure was generated using MATLAB software.

Based on the preparation phase in Fig. 7 (important stage in process mining) and the previous section (methodology), this research utilized raw data (in ASCII format) of local-regional earthquakes with a magnitude ≥ 4 that occurred between 1999 and 2018 in the region spanning 44–51° E and 38–42.5° N. The study area encompasses 388,111.5 km² (Fig. 5b) and includes a database of approximately 300 earthquake waveform signals.

These signals were recorded by 20 seismic stations equipped with both broadband and short-period sensors located in Armenia and Azerbaijan and its surrounding regions. The data collected were sourced from the U.S. Geological Survey (USGS) and the northwest seismic stations of the Iranian Seismological Center (IRSC).

**Fig. 6:**
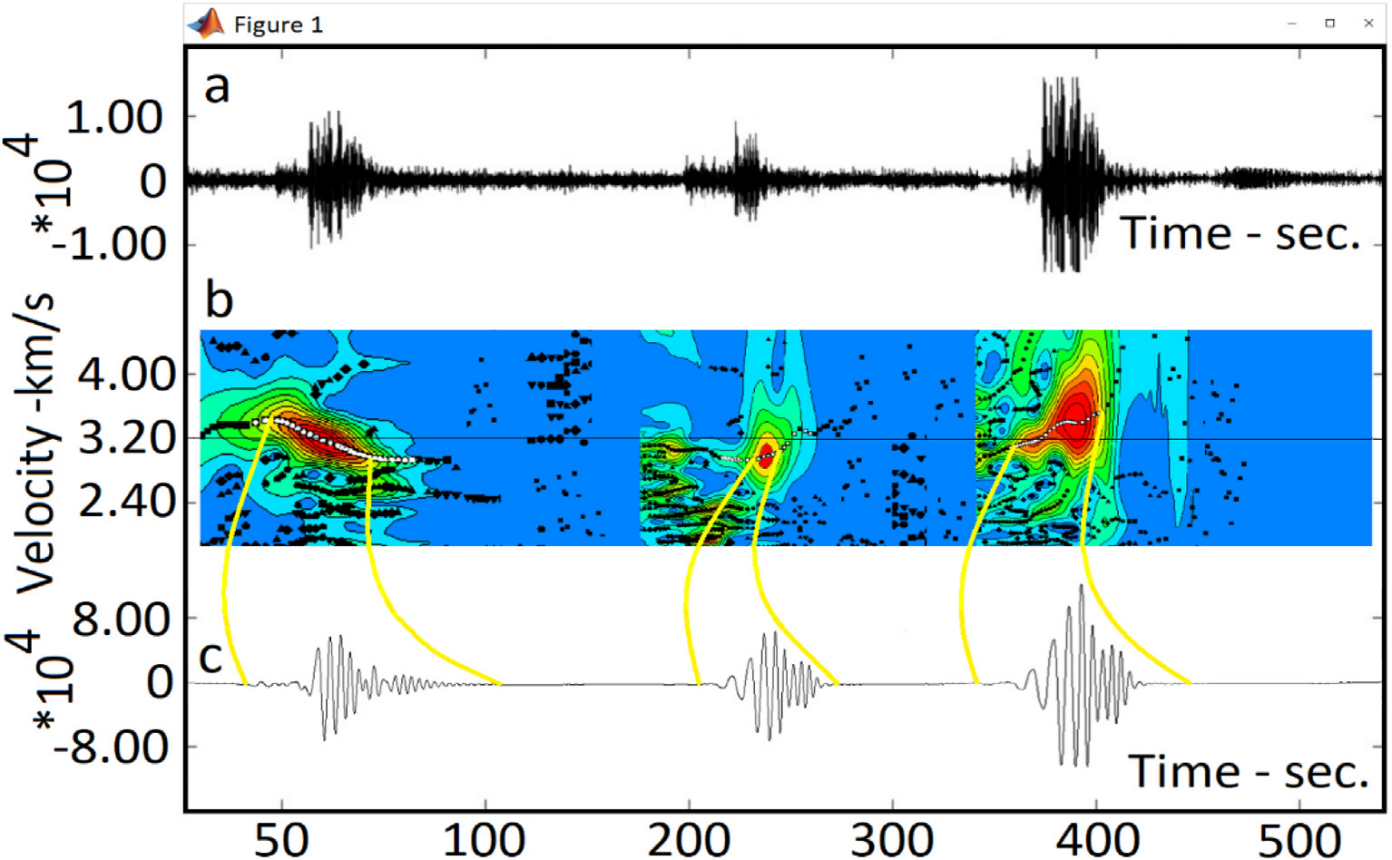
A sample of processed raw data: a. The raw data (signal) prior to processing and the application of the process mining algorithm; b. The energetic red region of the signal (often referred to as the fundamental mode). c. Cleaned signal. Sections b and c are derived from the implementation of process mining techniques and specialized computer codes aimed at optimizing of processing speed and data cleaning. This processing operation is performed on each data of this study. The figure was generated using MATLAB software.

During the process mining, a total of 900 quality signals were analyzed. An example of these signals is illustrated in Fig. 6. Each event consists of three components: east-west, north-south, and vertical.

(1) $$\left(4.5\;\times \;111\;\text{km}\right)=499.5\;\text{km}\;\&\;\left(7\;\times \;111\;\text{km}\right)\;=777\;\text{km}\;\Rightarrow 499.5\;\text{km}\;\times \;777\;\text{km}\;=\;388111.5\;km^{2}$$

(2) (300signals×20stations)=6000⇒6000×3components=18000components(data)

The regularization parameter α is employed to smooth the maps, while the parameter σ is used to set an acceptable error range for removing signal dispersions with high errors (Fig. 6-b). These parameters are utilized in specialized computer programs for database processing and preparation.

**Fig. 7:**
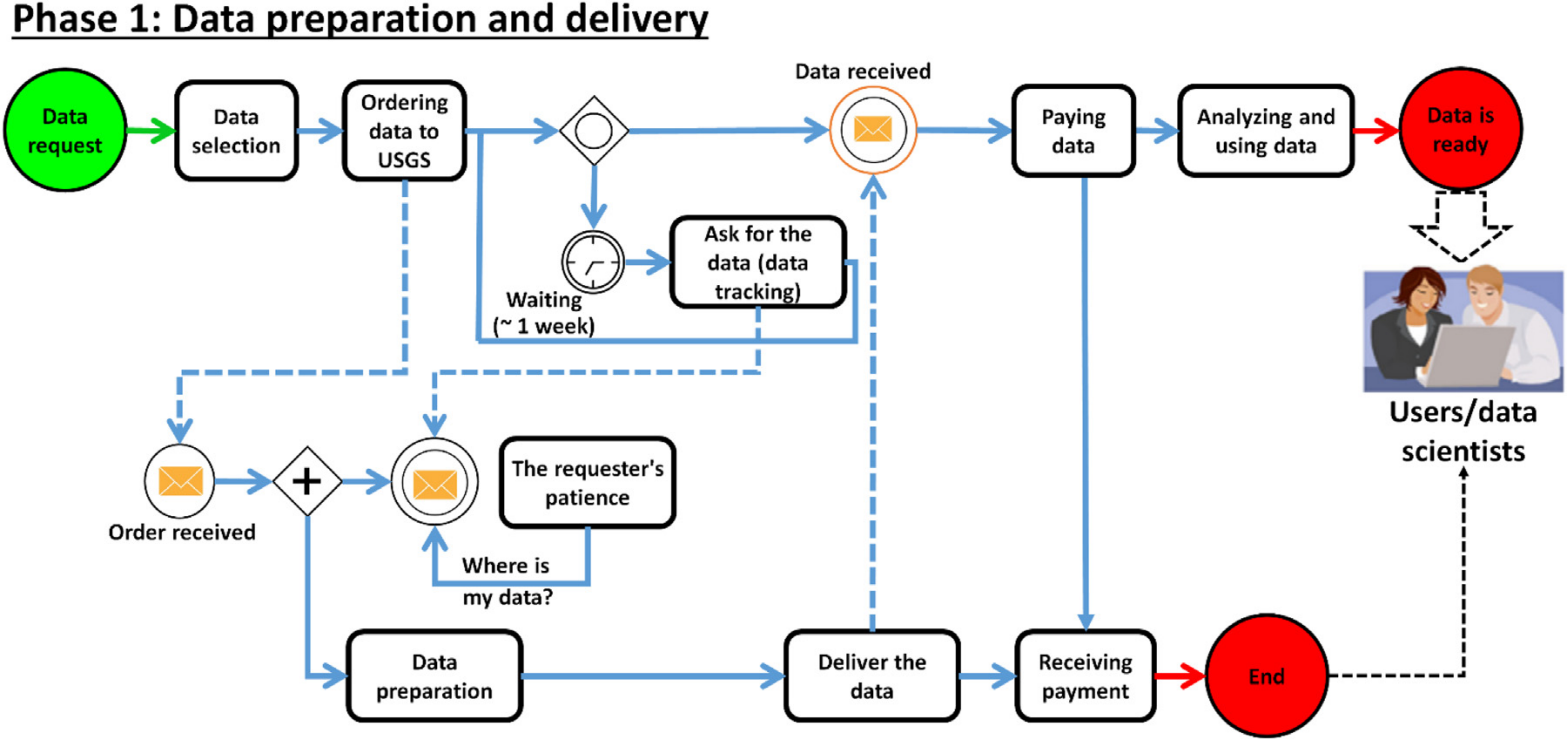
The preparation-to-delivery phase is the most important phase in process mining. It corresponds to Phase 1 of the BPMN model for data processing (production and delivery) at the Iranian Seismological Center (IRSC). Analyzing and identifying are very important in the steps of conducting the work, arranging, and choosing the appropriate symbols required for designing this model. As mentioned, the process of storing and delivering the data bank is nearly identical across seismic centers worldwide. The tools available at bpmn.io, Disco, and bpmn-io on GitHub were used to draw the graph. PowerPoint and MATLAB-Simulink software solutions were also employed to enhance the clarity of the images.

This type of data is collected and stored continuously, 24 h a day, by seismic networks globally and can be regarded as big data. The data mining process of big data covers several steps such as: database selection according to the research purpose; data extraction and integration (downloading the required data and combining data from multiple sources); data cleaning and transformation (removal of incorrect data, filing in missing data, generating new variables, converting data format, and ensuring data consistency); data mining (extraction of implicit relational patterns through traditional statistics or machine learning-ML); pattern evaluation (focuses on the validity parameters and values of the relationship patterns of the extracted data); and assessment of the results (translation of the extracted data-relationship model into comprehensible knowledge made available to the public). In the next section, this study focuses on analyzing data mining and process mining methods by detailing the algorithms and key steps in the data science process, following the procedures illustrated in Fig. 7 and the event logs presented in Table 2.

**Table 2:** A sample of list of the actions or event-logs in this study.

Case ID	Activities	Resources	Timestamp	Product	Prod-price	Quality	Address
2350	place order	**Amitis**	2018.01.02 02:29:35	IRIS	4 $	5	IUGT
2281	pay	**Negin**	2018.01.02 02:35:21	IRIS	4.5 $	4	IUGT
2257	Prepare delivery	**Nadia**	2018.01.02 02:45:20	IRIS	3.5 $	3.5	IUGT
2245	Prepare delivery	**Aiden**	2018.01.02 03:01::33	IRIS	4 $	4	IUGT
2272	Confirm payment	**Mahtab**	2018.01.02 03:05:11	IRIS	5 $	5	IUGT
2218	Confirm payment	**Mahtab**	2018.01.02 02:08:18	IRIS	4 $	4	IUGT
2245	Make delivery	**Kourosh**	2018.01.02 02:20:12	IRIS	3.5 $	3.5	IUGT
2272	pay	**Mahtab**	2018.01.02 02:35:36	IRIS	4 $	4	IUGT
…	**…**	**…**	**…**	**…**	**…**	**…**	**…**
Date range (Case id)		Activities (Activity name)	Cases (Timestamp)	Traces	Resources	Events	Other data
2017 01 – 2018–01		8	941	4	7	7528	–

## Results and discussion of process mining in this research

As mentioned, this study seeks to analyse: a) the data request from a data center, b) the steps involved in data preparation, and c) the delivery of a large database using process mining methods, in order to reduce the delivery time of the large database required by the Abry (PhdS) student In fact, by utilizing effective and efficient process mining diagrams in the creation and delivery of a database, the goal is to illustrate process efficiency, as well as to validate, model, and visualize processes based on stored-log entries.

To achieve this, a standardized and permissionless BPMN mark-up language is essential for modelling processes, designing workflows across the system, identifying bottlenecks, and detecting breakpoints. This approach facilitates the analysis, validation, modeling, and visualizing processes using process mining, as outlined in Fig. 7 and Table 1, Table 2.

In this research, Abry (PhDS) began to gather and processing a dataset from the IRSC for their PhD thesis. Receiving the massive database from the Iranian Seismological Center takes approximately two months (Fig. 7). In the best case scenario, the delivery of this database takes about 25 days. To enhance accuracy and save time, the Abry (PhDS) decided to apply process mining techniques at the start-up of the dataset. In this regard, several key steps need to be thoroughly examined as follows:

1. Extracting Data: Initially, the Abry (PhDS) collects event data from the IRSC e-commerce website. However, the student is unable to prepare the dataset independently and must reach out to the IRSC center for assistance. Event data includes information such as case ID, activity, and timestamp (e.g., on 08.02.2017 at 08:23:44; Abry (PhDS) placed an order with ID 2250 (Table 1, Table 2).

Also, eXtensible Event Stream (XES) is the standard format for process mining supported by the majority of process mining tools. XES was adopted in 2010 by the IEEE Task Force on Process Mining as the standard format for logging events. It has become an official IEEE standard in 2016 [10]. In this study, the scope of the process was chosen according to the Table 1and 2.

2. Process Discovery: Using the collected data, Abry (PhdS) applies process discovery algorithms to find the actual processes occurring inside start-up.
3. Conformance checking: Equipped with the process model, the Abry (PhdS) can replay the event data against it to determine if there are any discrepancies between the actual daily processes and the ideal processes outlined in the model.

**Fig. 8:**
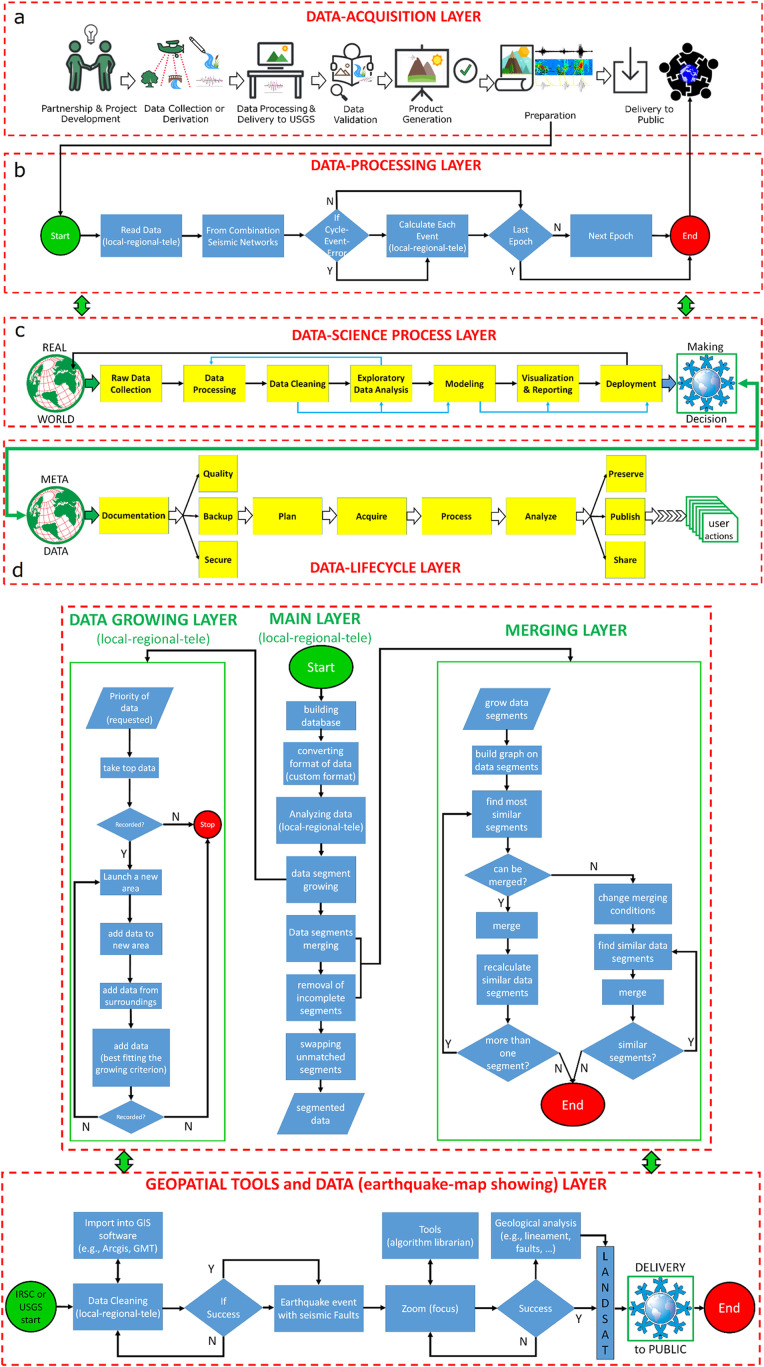

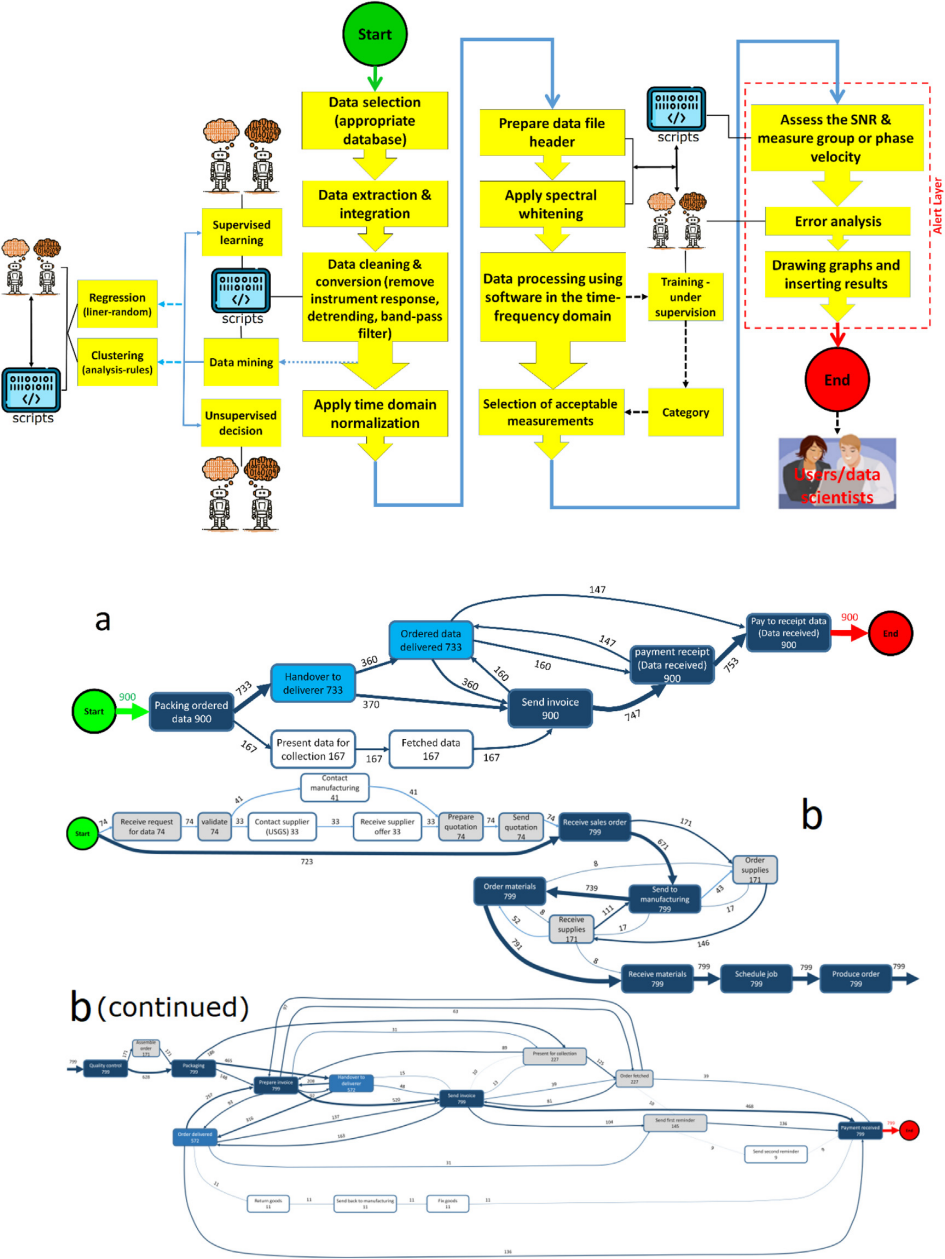
Schematic overview of the various layers in the database workflow and complete system flowchart from data acquisition to processing and validation: a. data acquisition layer; b. data processing flow chart layer; c. data science process layer; d. data lifecycle layer. The symbols used in the data acquisition layer were adapted with slight modifications by Abrehdari from the [https://www.usgs.gov] website. The tools available at bpmn.io, Disco, and bpmn-io on GitHub were used to draw the graph. PowerPoint and MATLAB-Simulink software solutions were also employed to enhance the clarity of the images. Fig. 8 provides a schematic representation of the machine learning-based data processing algorithm and framework for the database. The tools available at bpmn.io, Disco, and bpmn-io on GitHub were used to draw the graph. PowerPoint and MATLAB-Simulink software solutions were also employed to enhance the clarity of the images. Fig. 8 schematic representation of the manual data processing workflow during 25 days prior to implementing the process mining technique. The pseudocodes for the main scripts depicted in this figure has been included in the Supplementary Material section of the paper. PowerPoint was employed to enhance the clarity of the images. Fig. 8 Map of the process mining based on Table 2; a-b. The scope of the process Packaging-Contains encompasses the elements depicted in Fig. 7, along with its hidden operations (e.g., data mining, supervised learning, scripts, …). The tools available at bpmn.io, Disco, and bpmn-io on GitHub were used to draw the graph. PowerPoint and MATLAB-Simulink software solutions were also employed to enhance the clarity of the images.

Fig. 7, Fig. 8 depict the fundamental steps outlined above for the core layers of database acquisition. Figs. 8a-d respectively illustrate the fundamental layers of data acquisition within a database center. All layers continuously exchange information with each other around the clock.

The Supplementary Material section of the article contains well-structured and concise pseudocodes for the MATLAB scripts employed in this study. These structured and concise pseudocode versions of the MATLAB script are well-suited for inclusion in scientific research. They abstract implementation details while preserving the underlying logic and workflow. The websites [https://fluxicon.com/disco/], [https://github.com/bpmn-io], and [https://github.com/bpmn-io] were used to draw the graphs.

### The scope of the process mining

According to Table 2, the scope of the process is depicted in Fig. 8. In this study, process mining relies on the availability of an event-log, in which each event corresponds to a case, an activity, and a specific timestamp. An event-log can be seen as a collection of cases, with each case representing a trace or sequence of events.

Event data can originate from a diverse array of sources, including database system (e.g., patient data in a hospital), a comma-separated values (CSV) file or spreadsheet, a transaction log (e.g., a trading system), a business suite system (e.g., SAP, Oracle), a message log (e.g., from IBM middleware), and an open API providing data from websites or social media, and other sources. The event data for this study consist of a diverse array of seismic signal sources recorded continuously around the clock.

Fig. 8 shows that process modeling and process mapping are distinct, but related, methods of visualizing and analyzing system processes.

### The structure of the process mining

**Fig. 9:**
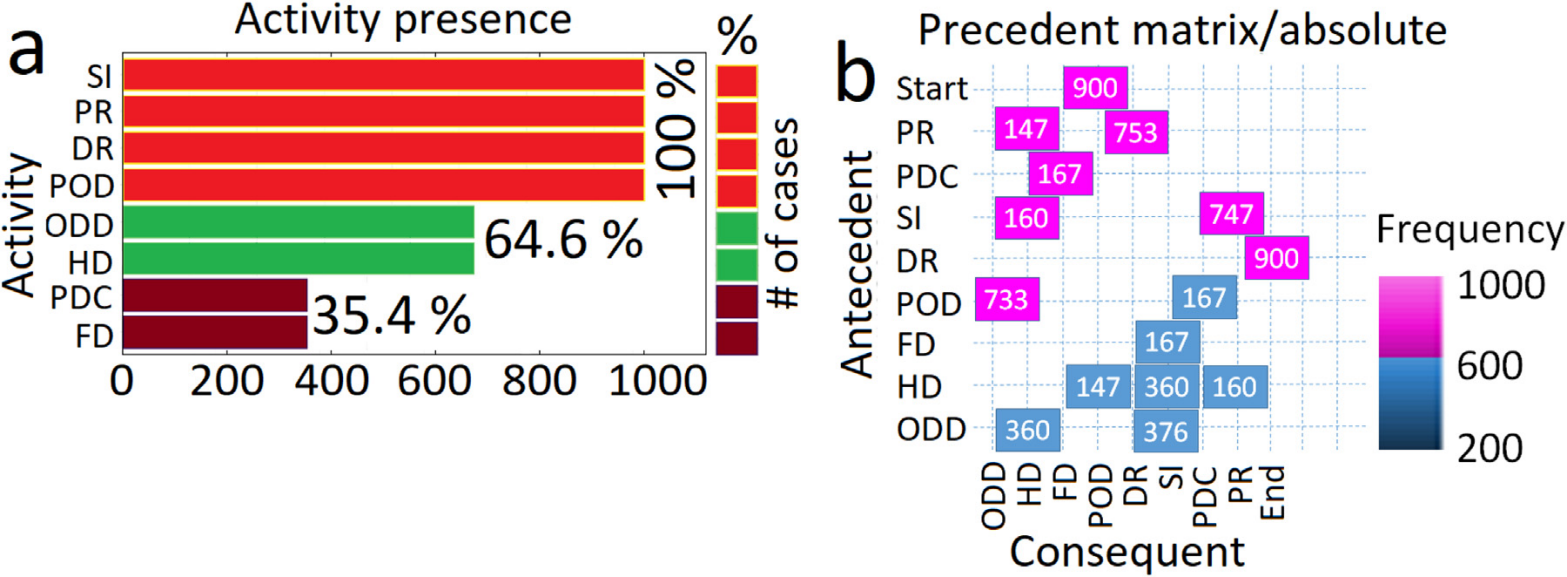
a. The graph illustrates the number of cases in which an activity takes place within the selected process. The % relative to the total number of cases is indicated at the right of the bars; b. The precedence matrix shows the sequences of activities. Abbreviations (e.g., PR, PDC, etc.) are illustrated in Fig. 8-a. The graph was drawn using both [https://fluxicon.com/disco/] and MATLAB software.

**Fig. 10:**
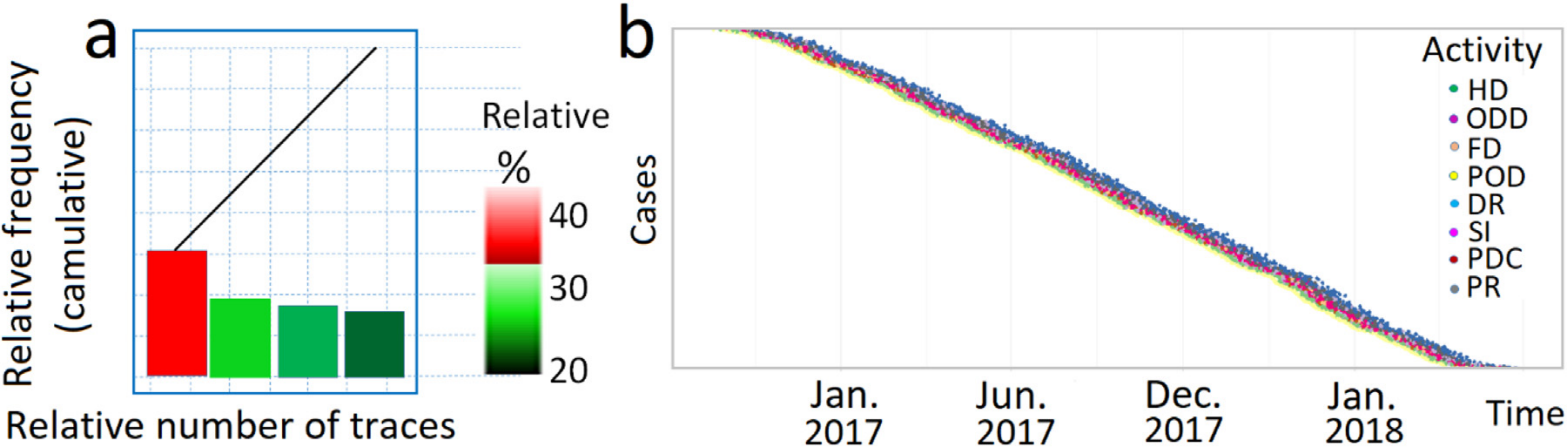
a. Traces-Sequence of activities; b. This chart displays all the cases on the y-axis, ordered by start date, while time is represented on the x-axis. Abbreviations (e.g., PR, PDC, etc.) are shown in Fig. 8-a. The graph was drawn using both [https://fluxicon.com/disco/] and MATLAB software.

In step 1 of process mining, some items are reviewed [for example, the Map (Fig. 8), Activities Presence (Fig. 9-a), Activities connexions (Fig. 9-b), Traces (Fig. 10-a), Dotted chart (Fig. 10-b), and Loops (there is no loop item in this process and so loop graph is not drawn)]. The diagrams presented in Figs. 9 through 13 represent the process mining map generated in this study and are subsequently analyzed and discussed.

Fig. 9-a illustrates activity repetitions —activities that occur multiple times within the process. If the graph does not display any data, it means that there are no repetitions in the process. There is also a distinction between repetitions and loops.

Activity presence, illustrated in Fig. 9-a, reflects a user's availability and current engagements on various platforms, particularly in communication and gaming settings. A repetition refers to an activity that occurs multiple times throughout the process, but not just after itself. A loop, in contrast, is an activity that happens directly after itself. You can specify whether the activity is a redo (performed by the same resource) or a repeat (carried out by someone else).

In Fig. 9-b, as can be seen which activity (antecedent) on the y-axis is followed by others activities (consequent) on the x-axis. The precedence matrix illustrates the sequences of activities within a process. It visually represents the relationships between activities, showing which ones must be completed before others can begin. This method helps in understanding dependencies, such as which activities are predecessors or successors to others, thereby facilitating effective project scheduling and management.

For example, the number shows the absolute frequency, which refers to the number of cases. It can also select the frequency relative to the antecedent, where the values total to 100 % of the antecedent activity. Similarly, the frequency relative to the consequent can be chosen, with the values totaling 100 % of the consequent activity.

In Fig. 10-a, the first graph displays the various sequences of activities in the process ordered by frequency, from the highest to the smallest. The 3 columns on the right represent, respectively, the frequency of the trace, the number of cases, and the cumulative frequency. The second graph visualizes the cumulative frequency as explained by the relative frequency. The line illustrates the percentage of cases attributed to each percentage of traces. We often observe a steep line at the beginning, indicating that 80 % of the cases (on the y-axis) are due to the 20 % of the most common traces (on the x-axis). Fig. 10-b offers information into the structure of the process and particular patterns, including the various sequences of activities, their corresponding frequencies, and any disruptions within the process.

As previously mentioned, the continuation of Fig. 10-b highlights activities that loop back on themselves, indicating that there is no actual loop in the process. In other words, these activities occur more than once in a row. You can select whether the activity is a redo (performed by the same resource) or a repeat (conducted by someone else). As a result, the percentage value of the items associated with the activities in the self-loop diagram is zero, and therefore, this diagram is not drawn.

### Resource involvement in the selected process

**Fig. 11:**
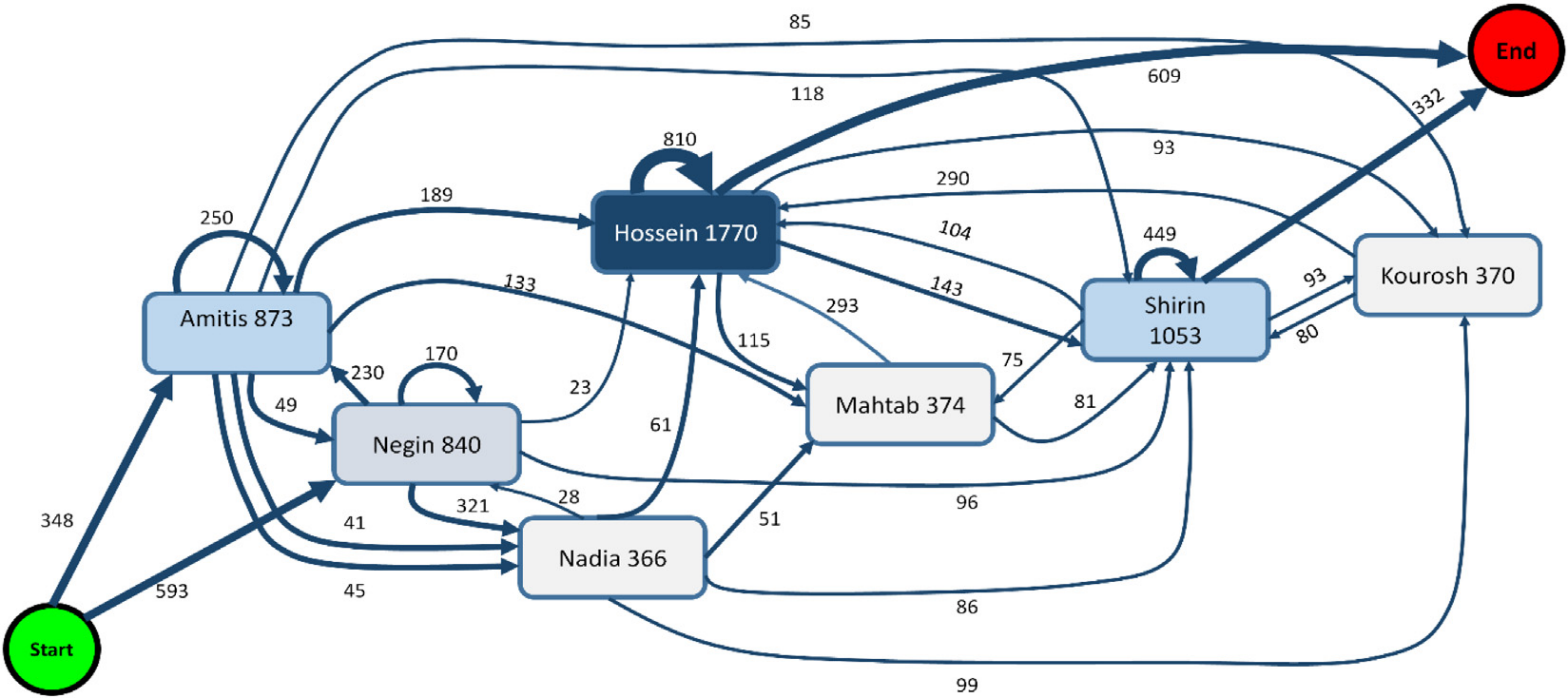
The resource involvement in the selected process corresponds with the map depicted in Fig. 8, which is discussed in section (3.1). The tools available at bpmn.io, Disco, and bpmn-io on GitHub were used to draw the graph. PowerPoint and MATLAB-Simulink software solutions were also employed to enhance the clarity of the images.

The diagrams at this stage are very similar to those illustrating resource involvement in the selected process in Section 3.2 (such as the Map, Activities, Involvement, Connections, and Loops figures). Therefore, these diagrams have not been included due to the study’s time-intensive nature and the number and similarity of the figures. However, the Map diagram (Fig. 11) and its Contains map (filtering type; Fig. 8b) have been created and are explained.

As previously mentioned, the frequency is associated with the resource, with numbers add up to 100 % for each resource. This includes the frequency spent on each resource based on activity or how activities are divided between resources. This has been excluded because of the study's time-consuming nature and the large number of similar Figures.

**Fig. 12:**
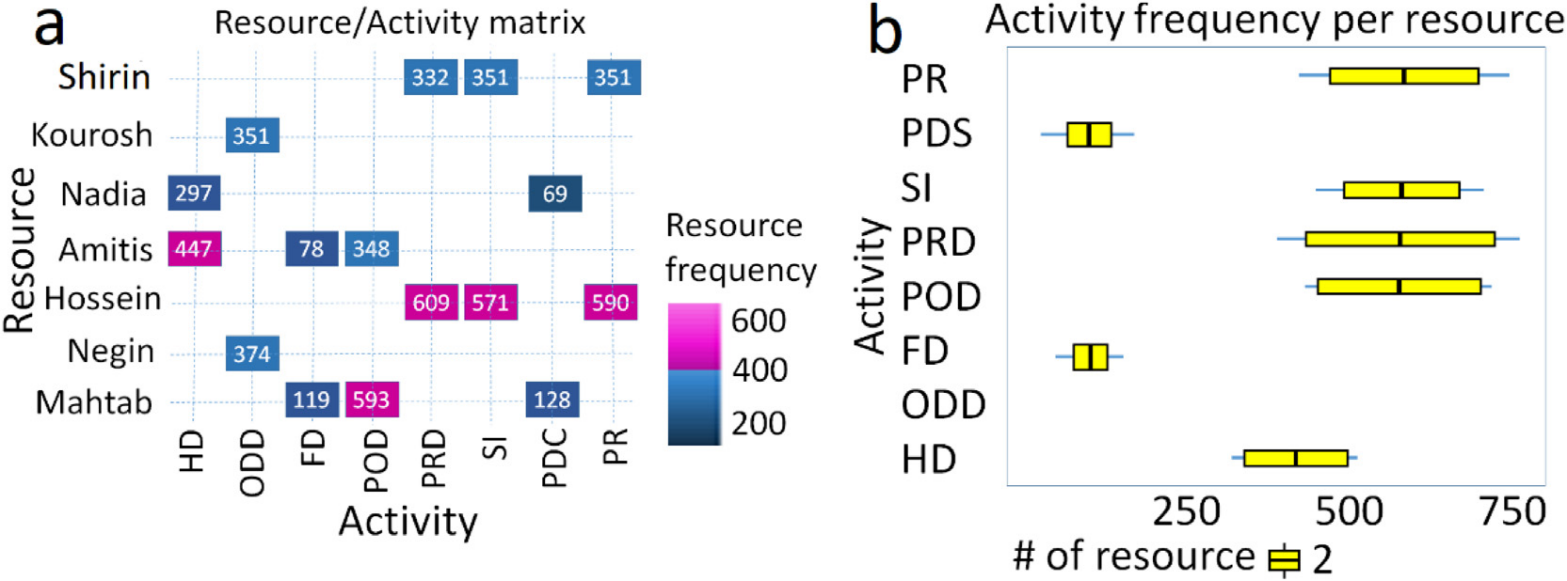
a. Resource-Activity Matrix graph shows who performs which activity; b. Activity frequency per resource. Abbreviations (e.g., PR, PDS, etc.) are illustrated in Fig. 8-a. The graph was drawn using both [https://fluxicon.com/disco/] and MATLAB software.

Fig. (12-a) illustrates the relationship between resource to activity or from activity to resource (row to column or vice versa). The number at the intersection of a user and an activity represents how many cases the resource allocated on this activity. The depiction and explanation of the percentage of resource-involvement and resource-repetition were omitted due to the study’s time-consuming nature and the large-number and similarity of the Figures. Fig. 12-b shows a boxplot of the distribution of the number of cases executed by different resources for a specific activity.

### Performance analysis step

**Fig. 13:**
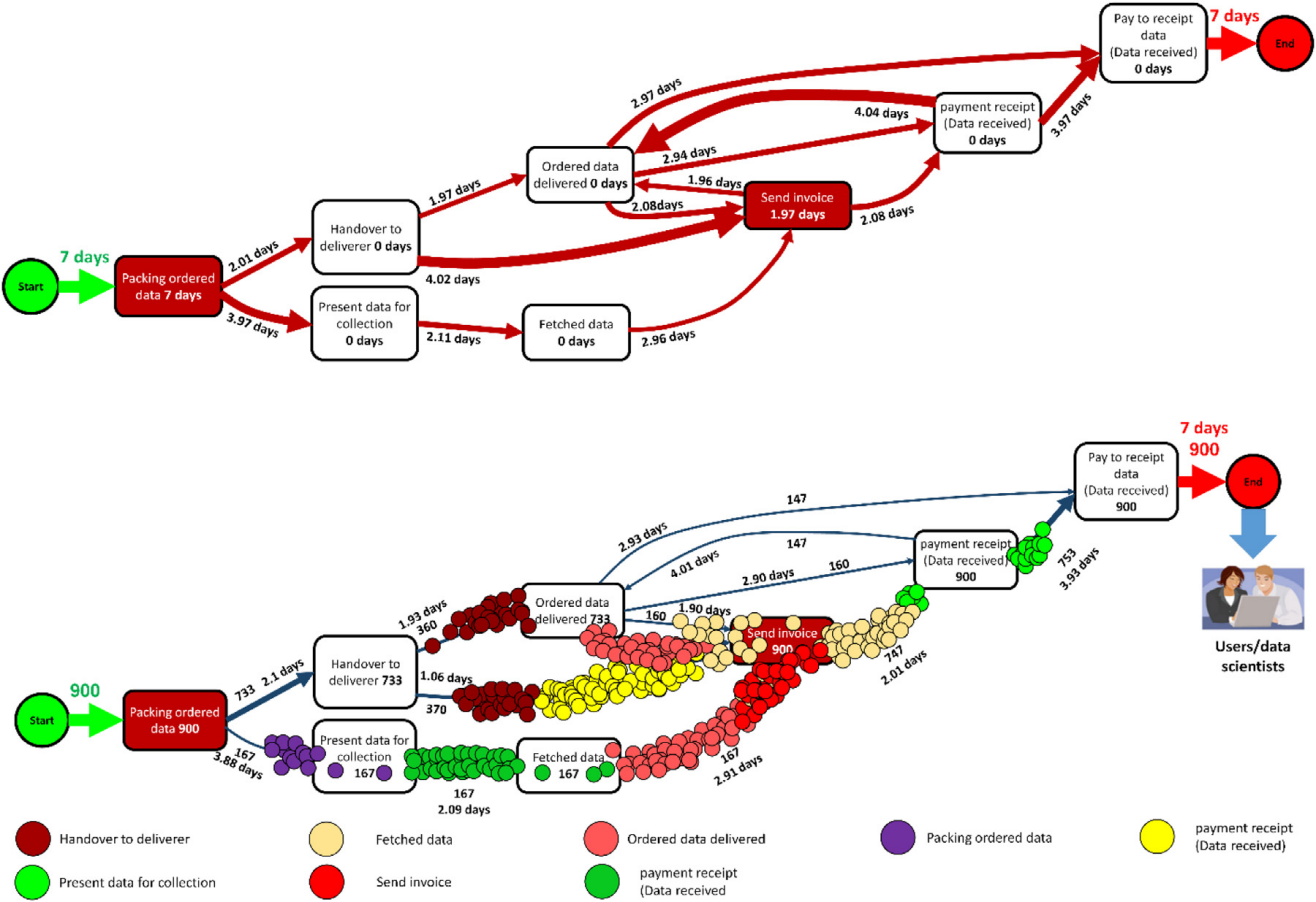
Performance analysis map (Metric for edges: Mean and Units for edges: days). Units can be selected as seconds, weeks, or hours, depending on the metric of interest. Fig. 13 performance analysis. The tools available at bpmn.io, Disco, and bpmn-io on GitHub were used to draw the graph. PowerPoint and MATLAB-Simulink software solutions were also employed to enhance the clarity of the images.

The Performance Analysis Map (Fig. 13) is beneficial for analysis the performance of the process. The metrics for both activities (processing time) and connections (idle time) are illustrated in the figure. For both the activities and connections categories, it is possible to choose the metric that best matches the usage method (the metric of interest). Note that if an activity consists only of an event, the throughput time will be zero. Processing time is only present when the activity includes two events, with a start and an end.

In Fig. 13, the process is replayed in relation to the start date during 25 days. This is insightful in different aspects: 1) the process is once again replayed with respect to the start date during 25 days. 2) The process allows visual inspection of process conformance to the theoretical model, visualization of process structure variability, and path complexity (how much the cases spread along the route). 3) It clearly identifies bottlenecks by displaying the map along with navigation, replay, motion readability, and zoom features.

**Fig. 14:**
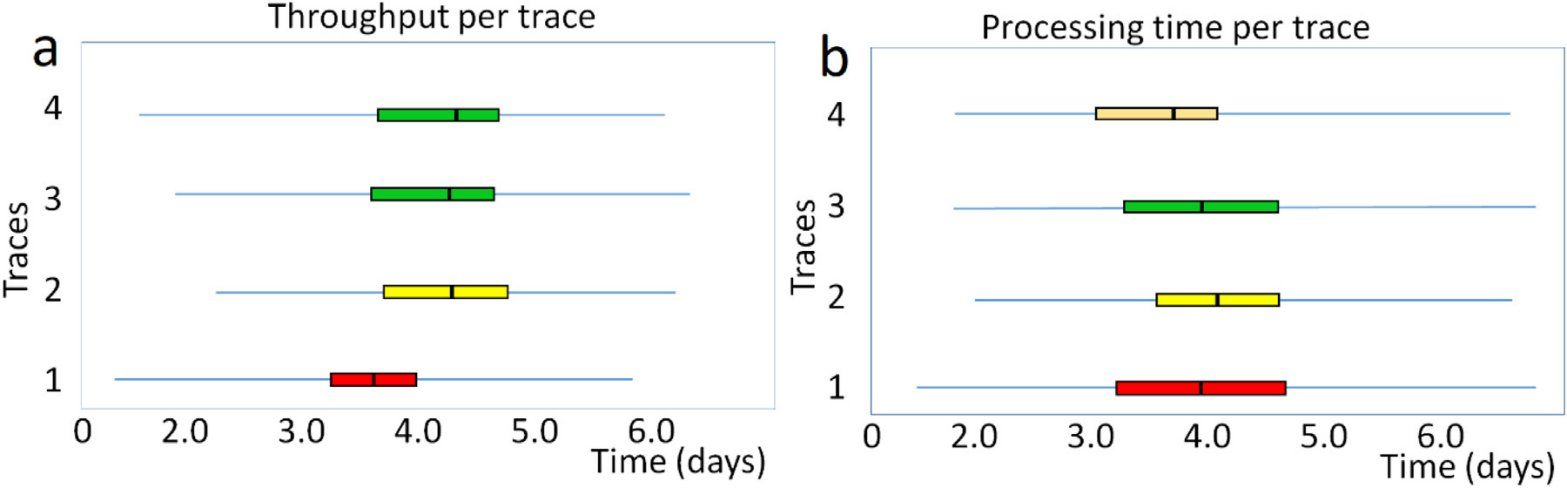
a. Performance analysis-throughput time; b. Processing time per trace or distribution of the processing time for each trace. The graph was drawn using both [https://fluxicon.com/disco/] and MATLAB software.

Fig. 14-a represents the throughput time of the process, i.e. the duration required to execute the process using a boxplot. It also enables checking throughput time at the trace level, broken down for each trace, or at the product level through throughput time decomposition for each trace. Throughput refers to the amount of material or items that pass through a system or process.

The processing time for the process is depicted in Fig. 14-b as a boxplot, representing how long it takes to execute the activities (not the transfer between ask). Thus, it is possible to look the data at the trace level, where processing time is broken for each trace, or at the activity level, where processing time broken down for each activity.

In fact, the box plot analysis comparing the processes before (Fig. 14-a) and after (Fig. 14-b) improvements in machine utilization is presented.

According to [11], machine utilization is a crucial performance indicator in manufacturing. This indicator is a ratio that measures the effective active time of a machine compared to the total time it is expected to be working. Failing to measure this variable can be a critical mistake for companies, as it directly impacts efficiency and productivity.

To interpret a box plot: The two ends of the segment (line) represent the minimum and maximum values. The box represents the interquartile range, which indicates the frequency between 25 %, 50 %, and 75 % of the traces/product have a throughput time in the range of the box. The line in the middle shows the median, meaning that 50 % of the traces/products have throughput times below this value and 50 % have throughput times above it. For example, the averages of each trace in Fig. (14-b) represent the following concepts:

Trace 1: Packing ordered data, handover to deliverer, ordered data delivered, send invoice, and pay to receipt data (Data received); mean= 3.98, median= 3.99, min= 1.62, max= 6.45, Q1 (25 %)= 3.19, Q3 (75 %)= 4.79, and The inter-quartile ranges (IQR)= 1.6 (IQR is a measure based on the quartiles of a dataset).

Trace 2: Packing ordered data, present data for collection, fetched data, send invoice, and pay to receipt data (Data received); mean= 4.07, median= 4.05, min= 1.53, max= 6.31, Q1 (25 %)= 3.6, Q3 (75 %)= 4.78, and IQR= 1.18.

Trace 3: Packing ordered data, handover to deliverer, send invoice, ordered data delivered, payment receipt (Data received), and Pay to receipt data (Data received); mean= 4.0, median= 4.01, min= 1.57, max= 6.48, Q1 (25 %)= 3.29, Q3 (75 %)= 4.66, and IQR= 1.38.

Trace 4: Packing ordered data, handover to deliverer, send invoice, payment receipt (Data received), ordered data delivered, and pay to receipt data (Data received); mean= 3.82, median= 3.87, min= 1.55, max= 6.28, Q1 (25 %)= 3.03, Q3 (75 %)= 4.69, and IQR= 1.66.

## Conclusion

This study aims to examine how process mining techniques can reduce the delivery time of data requests, as well as streamline the preparation and delivery process of big data in large-scale data centers. It examines process mining from a different and simple perspective and reveals its role in optimizing manual operations. The topic of applying process mining techniques to streamline the creation and delivery of seismic databases is both timely and innovative, particularly in an age where efficient management of large-scale scientific data is becoming increasingly vital.

This study introduces a novel and promising approach to reduce manual effort and turnaround time in seismological data handling, as well as in other comparable big-data production centers, and it has the potential to contribute meaningfully to both research and operational practices in this domain. This study’s concept can also support the advancement of new techniques focused on optimizing the time required for creating, storing, and delivering large-scale data.

## Ethics statements

Our research does not involve human subjects, animal experiments, or social media data.

## CRediT authorship contribution statement

**Seyed Hossein Abrehdari:** Writing – original draft, Software, Data curation, Visualization, Investigation, Validation, Supervision, Writing – review & editing.

## Declaration of competing interest

The authors declare that they have no known competing financial interests or personal relationships that could have appeared to influence the work reported in this paper.

## Appendix Supplementary materials

Image, application 1
