# Supplementary material for: Leveraging the process mining technique to optimize data preparation time in a database used as an automated data delivery center
Source: MethodsX. 2025 Jun 12;15:103428. doi: 10.1016/j.mex.2025.103428 (PMC12268689; doi:10.1016/j.mex.2025.103428)
Supplement: Supplementary file 1 [file mmc1.docx]

Below are the two main concise and structured pseudocode versions of the MATLAB script employed in this study. These pseudocodes streamline the implementation details while maintaining the essential logic and workflow.

BEGIN

cd C:\Users\userx\Desktop\Abrehdari\prog\final program

// Load data from 'RESULTS.DAT'

LOAD data from 'RESULTS.DAT' into res

// Extract x, y coordinates, and velocity from loaded data

x0 = first column of res

y0 = second column of res

vel = fourth column of res

// Create meshgrid from x0 and y0

[x, y] = MESHGRID(x0, y0)

// Interpolate velocity data onto the grid

[xi, yi, zi] = GRIDDATA(x0, y0, vel, x, y, 'linear')

// Create a figure

FIGURE(1)

// Create a pcolor plot of the interpolated data

z = PCOLOR(xi, yi, zi)

// Add a colorbar to the plot

COLORBAR

// Keep the current plot

HOLD ON

// Change directory to the folder containing fault files

CHANGE DIRECTORY to 'C:\Users\userx\Desktop\Abrehdari\prog\faults'

// Get a list of all files matching '*all_file'

pro = DIRECTORY LIST of files matching '*all_file'

// Loop through each file

FOR i = 1 to LENGTH(pro) DO

// Load the data from the current file

prv = LOAD(pro(i).name)

// Extract latitude and longitude from the loaded data

lat = second column of prv

lon = first column of prv

// Find indices where latitude is between 37 and 43, and longitude is between 38 and 50

l = FIND indices where lat >= 37 AND lat <= 43 AND lon >= 38 AND lon < 50

// Plot the filtered data

PLOT prv(l, 1) vs prv(l, 2) with black dots

END FOR

// Get a list of all files matching 'file*'

pro = DIRECTORY LIST of files matching 'file*'

// Loop through each file

FOR i = 1 to LENGTH(pro) DO

// Load the data from the current file

prv = LOAD(pro(i).name)

// Plot the data

PLOT prv(:, 1) vs prv(:, 2) with red lines

END FOR

END

BEGIN

// Initialization

Clear all variables and command window

Add program folder to path

Close all open files

Define base data path

// Prepare output directories

Create directory for path files

Change directory to dispersion data folder

// Define target periods and open output files for each period

Define periods = [5, 10, 15, 20, 25, 30, 35, 40, 45, 50, 55, 60, 65, 70]

For each period in periods:

Open output file PATH<period> for writing

// Loop over all subdirectories in dispersion folder

For each subdirectory (excluding '.' and '..'):

Change directory to subdirectory

If neither 'disp.d' nor 'exportdisp*' exists:

Return to parent directory and continue

// Load dispersion data

If 'disp.d' exists:

Read period and velocity from 'disp.d'

Else:

Load period and velocity from 'exportdisp*'

Round period values down to nearest integer

// For each target period

For each period in periods:

Find indices where period matches current target period

If no match or velocity at first index >= 4:

Continue to next period

// Read SAC file for event and station coordinates

Load SAC file

If station longitude > 38:

Write event latitude, event longitude, station latitude, station longitude, velocity, and flag to corresponding PATH<period> file

Return to parent directory

Close all output files

// Generate ray path files for each period

Change directory to path files folder

For each directory starting with 'Per':

Change into directory

Open 'ray_path' file for writing

Load 'PATHS' file

For each row in 'PATHS':

Write event and station coordinates in sequence, separated by delimiter

Close 'ray_path' file

Return to parent directory

// Write parameter file

Open 'param' file for writing

Write predefined grid and analysis parameters

Close 'param' file

// Run external processing for each period

For each PATH* file:

Create new directory for period

Copy PATH* file and 'param' file into new directory

Change into new directory

// Calculate ray density for each period

For each directory starting with 'Per':

Change into directory

Open 'density_path' file for writing

Load 'PATHS' data (event and station coordinates)

// Define grid boundaries and step sizes

Set latitude and longitude min/max

Set grid step sizes

// Loop over grid blocks

For each longitude block:

For each latitude block:

Define current grid cell

Calculate rays crossing current grid cell using which_line function

Write longitude, latitude, and number of rays to 'density_path' file

Close 'density_path' file

Return to parent directory

END
